# A small peptide antagonist of the Fas receptor inhibits neuroinflammation and prevents axon degeneration and retinal ganglion cell death in an inducible mouse model of glaucoma

**DOI:** 10.1186/s12974-019-1576-3

**Published:** 2019-09-30

**Authors:** Anitha Krishnan, Andrew J. Kocab, David N. Zacks, Ann Marshak-Rothstein, Meredith Gregory-Ksander

**Affiliations:** 1 Department of Ophthalmology, The Schepens Eye Research Institute, Massachusetts Eye and Ear, Harvard Medical School, 20 Staniford Street, Boston, MA USA; 2 ONL Therapeutics, Ann Arbor, MI USA; 3 Department of Ophthalmology and Visual Sciences, University of Michigan, Ann Arbor, MI USA; 4 Department of Medicine, University of Massachusetts Medical School, Worcester, MA USA

**Keywords:** Glaucoma, Neuroprotection, Retinal ganglion cell, Fas ligand, Fas receptor, Apoptosis, Neuroinflammation, Neurodegeneration, Microglia, Immune privilege

## Abstract

**Background:**

Glaucoma is a complex, multifactorial disease where apoptosis, microglia activation, and inflammation have been linked to the death of retinal ganglion cells (RGCs) and axon degeneration. We demonstrated previously that FasL-Fas signaling was required for axon degeneration and death of RGCs in chronic and inducible mouse models of glaucoma and that Fas activation triggered RGC apoptosis, glial activation, and inflammation. Here, we investigated whether targeting the Fas receptor with a small peptide antagonist, ONL1204, has anti-inflammatory and neuroprotective effects in a microbead-induced mouse model of glaucoma.

**Methods:**

Intracameral injection of microbeads was used to elevate intraocular pressure (IOP) in Fas-deficient (Fas^lpr^) mice and WT C57BL/6J mice that received an intravitreal injection of the Fas inhibitor, ONL1204 (2 μg/1 μl) (or vehicle only), on day 0 or day 7 after microbead injection. The IOP was monitored by rebound tonometry, and at 28 days post-microbead injection, Brn3a-stained RGCs and paraphenylenediamine (PPD)-stained axons were analyzed. The effects of ONL1204 on retinal microglia activation and the expression of inflammatory genes were analyzed by immunostaining of retinal flatmounts and quantitative PCR (qPCR).

**Results:**

Rebound tonometry showed equivalent elevation of IOP in all groups of microbead-injected mice. At 28 days post-microbead injection, the RGC and axon counts from microbead-injected Fas^lpr^ mice were equivalent to saline-injected (no IOP elevation) controls. Treatment with ONL1204 also significantly reduced RGC death and loss of axons in microbead-injected WT mice when compared to vehicle-treated controls, even when administered after IOP elevation. Confocal analysis of Iba1-stained retinal flatmounts and qPCR demonstrated that ONL1204 also abrogated microglia activation and inhibited the induction of multiple genes implicated in glaucoma, including cytokines and chemokines (GFAP, Caspase-8, TNFα, IL-1β, IL-6, IL-18, MIP-1α, MIP-1β, MIP-2, MCPI, and IP10), components of the complement cascade (C3, C1Q), Toll-like receptor pathway (TLR4), and inflammasome pathway (NLRP3).

**Conclusions:**

These results serve as proof-of-principal that the small peptide inhibitor of the Fas receptor, ONL1204, can provide robust neuroprotection in an inducible mouse model of glaucoma, even when administered after IOP elevation. Moreover, Fas signaling contributes to the pathogenesis of glaucoma through activation of both apoptotic and inflammatory pathways.

## Background

Glaucoma is the leading cause of irreversible blindness worldwide, characterized by the progressive loss of retinal ganglion cells (RGCs). A recent study estimates that approximately 60 million people worldwide currently suffer from glaucoma, and with the rapidly growing aging population, this number is predicted to exceed 100 million by 2040 [1]. Elevated intraocular pressure (IOP) is a major risk factor for the development of glaucoma, and lowering IOP remains the only treatment for this disease [2]. However, the continued progression of the disease in some patients despite successful reduction of IOP [3], combined with the increasing incidence of normal-tension glaucoma [4, 5] and the absence of neurodegeneration in some patients with elevated IOP [6], indicates that IOP-independent mechanisms contribute to the initiation and progression of glaucoma. Therefore, a current priority in the glaucoma field is to further define the molecular mechanisms of RGC death and axon degeneration in order to develop IOP-independent therapeutic treatment strategies to halt disease progression and preserve vision.

There is substantial evidence that apoptosis of RGCs is the final common pathway in both human and experimental models of glaucoma [7–10]. However, using the DBA/2J mouse model of spontaneous glaucoma, Libby et al. demonstrated that genetic ablation of the proapoptotic molecule BCL2-associated X protein (BAX) prevents apoptosis of RGCs, but does not prevent axon degeneration [11]. Similarly, McKinnon et al. demonstrated that gene therapy with a potent caspase inhibitor, baculoviral IAP repeat-containing protein-4 (BIRC4), only protects about 50% of the RGCs and optic nerve axons in a rodent model of elevated IOP [12]. Therefore, while RGC apoptosis is the common endpoint in glaucoma, therapeutic approaches that only target the apoptotic pathway in RGCs do not completely prevent glaucomatous neurodegeneration.

Glaucoma is a complex multifactorial disease, and while the exact molecular mechanisms of RGC apoptosis are not completely understood, there is growing evidence that suggests microglia activation and neuroinflammation play a central role in both early and late stages of glaucomatous neurodegeneration [13–16]. In human and experimental models of glaucoma, activated microglia are detected in the optic nerve head (ONH) and retina [14–19] and the extent of microglia activation correlates with the extent of neurodegeneration [20, 21]. Moreover, blocking microglia activation with minocycline [14, 20] or anti-TNFα [22, 23] prevents the infiltration of immune cells and significantly reduces axon degeneration and death of RGCs in experimental models of glaucoma. Together, these data suggest that activated microglia are the driving force behind glaucomatous neurodegeneration. However, the molecular mechanism(s) that mediate microglia reactivity in glaucoma are not well understood.

Fas ligand (FasL) is a type II transmembrane protein of the TNF family, which is best known for its ability to induce apoptosis upon binding to the Fas receptor [24–27]. However, we demonstrated that within the eye, FasL can be expressed as a membrane-bound protein (mFasL), which is pro-apoptotic and pro-inflammatory, or it can be cleaved and released as a soluble isoform (sFasL), which is non-apoptotic and non-inflammatory [28–30]. In the normal immune-privileged eye, where inflammation is tightly regulated, FasL is primarily expressed as the non-apoptotic, non-inflammatory sFasL [31]. However, in the DBA/2J mouse model of glaucoma, a shift in the expression of FasL from the soluble form to the proapoptotic and proinflammatory membrane form coincides with the loss of immune privilege and the development of glaucoma [31, 32]. These data suggest mFasL activation of the Fas receptor plays a central role in the pathogenesis of glaucoma. Moreover, treating mice with sFasL, via intravitreal adeno-associated virus-mediated gene delivery, provided significant neuroprotection of RGCs and axons, and this protection correlated with inhibition of retinal glial activation and the induction of the proinflammatory mediators [31]. These data demonstrate that blocking mFasL activation of the Fas receptor inhibits three hallmarks of glaucomatous degeneration: microglia activation, inflammation, and apoptosis. Therefore, we hypothesized that specifically blocking the Fas receptor with a small peptide inhibitor could serve as a novel neuroprotective approach in the treatment of glaucoma.

When developing a small peptide inhibitor of Fas, we first looked into the reports that Met, a growth factor receptor tyrosine kinase, could directly bind to and sequester the Fas receptor in hepatocytes [33]. This sequestration of the Fas receptor prevents Fas activation and subsequent apoptosis, identifying Met as an inhibitor of the Fas pathway. Using this information, we developed Met12, which is a small peptide that inhibits Fas-induced caspase-8 activation in the 661W photoreceptor cell line [34], for ocular use. In vivo, Met12 significantly inhibited photoreceptor apoptosis in a mouse model of retinal detachment [34]. More recently, we demonstrated that Met12 also inhibits Fas activation and subsequent apoptosis of photoreceptors and RPE in a sodium iodate-induced mouse model of retinal degeneration [35]. Together, these studies demonstrate that Met12 can be used in vivo to inhibit Fas-mediated apoptosis in models of retinal injury and degeneration.

Herein, we used a well-defined microbead-induced mouse model of elevated IOP to (i) examine the ability of a new derivative of Met12, ONL1204, to protect RGCs and prevent axon degeneration, and (ii) test the hypothesis that the Fas signaling pathway mediates microglia activation and the induction of neurodestructive inflammation in glaucoma. Our results demonstrate that a single intravitreal administration of the Fas inhibitor, ONL1204, significantly reduced RGC death and axon degeneration, even when administered after elevated IOP. Moreover, the neuroprotection correlated with significant inhibition of retinal microglia activation and inflammatory genes expression, suggesting that Fas signaling contributes to the pathogenesis of glaucoma through both apoptotic and inflammatory pathways. Together, these data underscore the value of targeting Fas in glaucoma and provide proof-of-principal that the small peptide inhibitor of the Fas receptor, ONL1204, can provide robust neuroprotection in an inducible mouse model of glaucoma, even when administered after elevated IOP.

## Materials and methods

### Animals

All animal experiments were approved by the Institutional Animal Care and Use Committee at Schepens Eye Research Institute and were performed under the guidelines of the Association of Research in Vision and Ophthalmology (Rockville, MD). The 8-week-old C57BL/6J WT mice (Stock No: 000664) and B6.MRL-Fas^lpr^/J Fas receptor-deficient mice (Stock No: 000482) were purchased from Jackson Laboratories (Bar Harbor, ME) and housed and maintained under cyclic light (12 L-30 lux: 12D) conditions in an AAALAC-approved animal facility at the Schepens Eye Research Institute. To avoid sex bias, equal numbers of male and female mice were included in each experimental group.

### Microbead-induced model of elevated IOP

Mice were anesthetized by intraperitoneal injection of a mixture of ketamine (100 mg/kg; Ketaset; Fort Dodge Animal Health, Fort Dodge, IA) and xylazine (9 mg/kg; TranquiVed; Vedco, Inc., St. Joseph, MO) supplemented by topical application of proparacaine (0.5%; Bausch & Lomb, Tampa, FL). Elevation of IOP was induced unilaterally by injection of polystyrene microbeads (FluoSpheres; Invitrogen, Carlsbad, CA; 15-μm diameter) into the anterior chamber of the right eye of each animal under a surgical microscope, as previously reported [31]. Briefly, microbeads were prepared at a concentration of 5.0 × 10^6^ beads/ml in sterile physiologic saline. The right cornea was gently punctured near the center using a sharp glass micropipette (World Precision Instruments Inc., Sarasota, FL). A small volume (2 μL) of microbeads was injected through this preformed hole into the anterior chamber followed by injection of an air bubble via the micropipette connected with a Hamilton syringe. Any mice that developed signs of inflammation (clouding of the cornea, edematous cornea, etc.) were excluded from the study.

### IOP measurements

IOP was measured with a rebound TonoLab tonometer (Colonial Medical Supply, Espoo, Finland), as previously described [31, 33]. Mice were anesthetized by 3% isoflurane in 100% oxygen (induction) followed by 1.5% isoflurane in 100% oxygen (maintenance) delivered with a precision vaporizer. IOP measurement was initiated within 2 to 3 min after animals lost toe pinch reflex or tail pinch response. Anesthetized mice were placed on a platform, and the tip of the pressure sensor was placed approximately 1/8 in. from the central cornea. Average IOP was displayed automatically after six measurements after elimination of the highest and lowest values. This machine-generated mean was considered as one reading, and six readings were obtained for each eye. All IOPs were taken at the same time of day (between 10:00 and 12:00 h) due to the variation of IOP throughout the day.

### Intravitreal injections

The intravitreal injections, just posterior to the limbus and parallel to the conjunctival vessels, were performed as previously described [31, 36]. Mice received a 1-μl intravitreal injection containing ONL1204 (2 mg/ml) or vehicle control on day 0 (just prior to injection of microbeads) or day 7 after microbeads injection.

### Quantification of optic nerve axons

For quantification of axons, optic nerves were dissected and fixed in Karnovsky’s reagent (50% in phosphate buffer) overnight. Semi-thin cross-sections of the nerve were taken at 1.0 mm posterior to the globe and stained with 1% p-phenylenediamine (PPD) for evaluation by light microscopy. Ten non-overlapping photomicrographs were taken at × 100 magnification covering the entire area of the optic nerve cross-section. Using ImageJ software, a 50 μM × 50 μM square was placed on each × 100 image and all axons within the square (0.0025 mm^2^) were counted using the threshold and analyze particles function in image J as previously described [31]. The average axon counts in the 10 images were used to calculate the axon density per square millimeter of the optic nerve (ON). Individuals blinded to the experimental groups performed all axon counts.

### Immunohistochemistry-retinal flat mount

Immediately following euthanasia, eyes were enucleated and fixed in 4% paraformaldehyde for 2 h at room temperature. The retina was detached from the eyecup, and four radial incisions reaching approximately 2/3 of the radius of the retina were made to create a butterfly shape. Retinal flatmounts were washed with PBS/T (0.1% Triton X-100) and permeabilized with 0.1% Triton X-100 in 20% superblock blocking buffer (2 ml superblock (Thermo Fisher cat no- 37580) + 8 ml PBS/T + 10μl Triton X) for 30 min at room temperature. Following permeabilization, the retinas were blocked in blocking solution (20% superblock + 10% goat serum) for 1 h at room temperature. Retinas were then incubated at 4 °C overnight with a primary Ab against Brn3a, a RGC-specific marker (Millipore Cat no- 1585, Billerica, MA), or against Iba1, a microglia/macrophage marker (Wako, Chemicals USA, Inc. Cat# 019-19741). An Alexa Fluor 555—conjugated for Brn3a— and Alexa Fluor 488—conjugated for IBA1 (Invitrogen)—was used as secondary Ab. Nuclei were counterstained with DAPI (vector stain).

### Quantification of retinal ganglion cells

To quantitate retinal ganglion cells, × 60 oil immersion was used and 16 non-overlapping images were taken (4–5 images per quadrant) using the × 60 oil immersion objective of Leica TCS SP5 confocal microscope system. All Brn3a-stained RGCs were quantitated using an automated counting platform we previously developed using CellProfiler software [37]. ImageJ software was used to calculate the area of each image, and the average number of RGCs in the 16 images was used to calculate the RGC density per square millimeter of retina. Individuals blinded to the experimental groups performed all RGC counts.

### Quantification of retinal microglia

To quantitate Iba1+ microglia/macrophages, image stacks of retinal flatmounts were acquired using the × 20 oil immersion objective (zoom 1.7, 35 μm depth (includes GCL and IPL)) of the Leica TCS SP5 confocal microscope system. The retina was divided into four quadrants, and one mid-peripheral region was imaged per quadrant for a total of four images per retina (480 μm by 480 μm per region). Microglial cells were counted manually by an individual blinded to the treatment groups using ImageJ software as previously described [38]. The longest cell process length, which is a marker of cell quiescence, was used as a morphometric descriptor to analyze microglia activation using NeruonJ Fiji Plugin as previously described [39]. Individuals blinded to the experimental groups performed all microglia quantification.

### Quantitative RT-PCR

RNA was isolated from the neural retina using a QIAGEN RNeasy Mini Kit (catalog number 74104), according to the manufacturer’s protocol. RNA was treated with DNase (catalog number AM222; Invitrogen) to ensure no contamination of genomic DNA. A total of 500 ng of RNA was reverse-transcribed (Thermo fisher Cat no 11756050 Superscript IV VILO master mix) according to the manufacturer’s instructions. cDNA was diluted 1:4 and then used for each amplification reaction. cDNA was treated with RNase H (18021-014; Invitrogen) to ensure the absence of ssRNA. Quantitative PCR (qPCR) reactions were performed in a 10 μl total volume using the FastStart Universal SYBR Green Master (Rox) (4913914001; Sigma) according to the manufacturer’s protocol. PCR cycles consisted of a denaturation step at 95 °C for 10 min, followed by 50 cycles of 95 °C for 15 s and 60 °C for 60 s. Each sample was subjected to melting curve analysis to confirm amplification specificity. Samples were run in duplicate, and each experiment included nontemplate control wells. Samples were normalized to house-keeping genes and expressed as the relative expression using the δ-delta Ct method. Relative expression to two house-keeping genes β2 microglobulin and PPIA was quantified using the formula: relative expression δ-delta CT = 2^(avg. gene cT−avg. saline-treated cT). Fold changes were calculated with respect to saline-injected control eyes. All of the primers used are listed in Table 1.

**Table 1:** RNA primers used for qPCR

Primers	Forward sequence (5′-3′)	Reverse sequence (5′-3′)
B2Micr	ACGTAACACAGTTCCACCCG	CGGCCATACTGGCATGCTTA
Ppia	GAGCTGTTTGCAGACAAAGTTC	CCCTGGCACATGAATCCTGG
C1Q	CAAGGACTGAAGGGCGTGAA	CAAGCGTCATTGGGTTCTGC
C3	AGCAGGTCATCAAGTCAGGC	GATGTAGCTGGTGTTGGGCT
Casp 8	CTCCGAAAAATGAAG GACAGA	CGTGGGATAGGATACAGCAGA
GFAP	CCCTGGCTCGTGTGGATTT	GACCGATACCACTCCTCTGTC
IL-1β	GAAATGCCACCTTTTGACAGTG	TGGATGCTCTCATCAGGACAG
IL-6	GTCCGGAGAGGAGACTTCAC	CTGCAAGTGCATCATCGTTGT
IL-18	AGTGAACCCCAGACCAGACT	TCAGGTGGATCCATTTCCTCAA
IP-10	TGAGCTAGGGAGGACAAGGA	GGA TGG CTG TCC TAG CTC TG
MCP1	GGCGGTCAAAAAGTTTGCCT	TTCTTCCGTTGAGGGACAGC
MIP-1α	TGCCAAGTAGCCACATCGAG	TGACCAACTGGGAGGGAGAT
MIP-1β	GTCCTTGCTCCTCACGTTCA	CCATCTCCATGGGAGACACG
MIP-2	GGCGGTCAAAAAGTTTGCCT	TTCTTCCGTTGAGGGACAGC
NLRP 3	ATTACCCGCCCGAGAAAGG	TCGCAGCAAAGATCCACACAG
TLR4	CTCTGGGGAGGCACATCTTC	CCCAGGTGAGCTGTAGCAT
TNF-α	CCTCTCATGCACCACCATCA	GCATTGCACCTCAGGGAAGA

### Survival assay

The ability of ONL1204 to inhibit FasL-mediated apoptosis of murine A20 B lymphoma cells was evaluated in vitro. Microvesicle preparations were isolated from transfected Neuro2a cells that expressed either murine mFasL (mFasL VP) or the vector control (Neo VP) as described previously [40]. A20 lymphoma cells were incubated for 4 h with titrations of ONL 1204 or the vehicle control together with a 1:100 dilution of either mFasL VP or Neo VP and then cultured overnight in the presence of ^3^H-thymidine. Survival was assessed by ^3^H-thymidine incorporation using the formula (cpm of mFasL VP + ONL1204 or vehicle)/cpm Neo VP + ONL1204 or vehicle).

### Statistics

Graph Pad Prism 8 (La Jolla, CA, USA) was used to perform statistical analysis of the data. For the A20 in vitro study, one-way ANOVA and Dunnett’s multiple comparisons test were used to compare different treatment groups. One-way ANOVA and Dunnett’s multiple comparisons test were used for RGC, axon, microglia, and qPCR analyses. Two-way ANOVA and Dunnett’s multiple comparisons test were used for all IOP comparisons. A *P* value of less than 0.05 was considered significant.

## Results

### ONL1204 blocks FasL-induced apoptosis of Fas+ targets

**Fig. 1:**
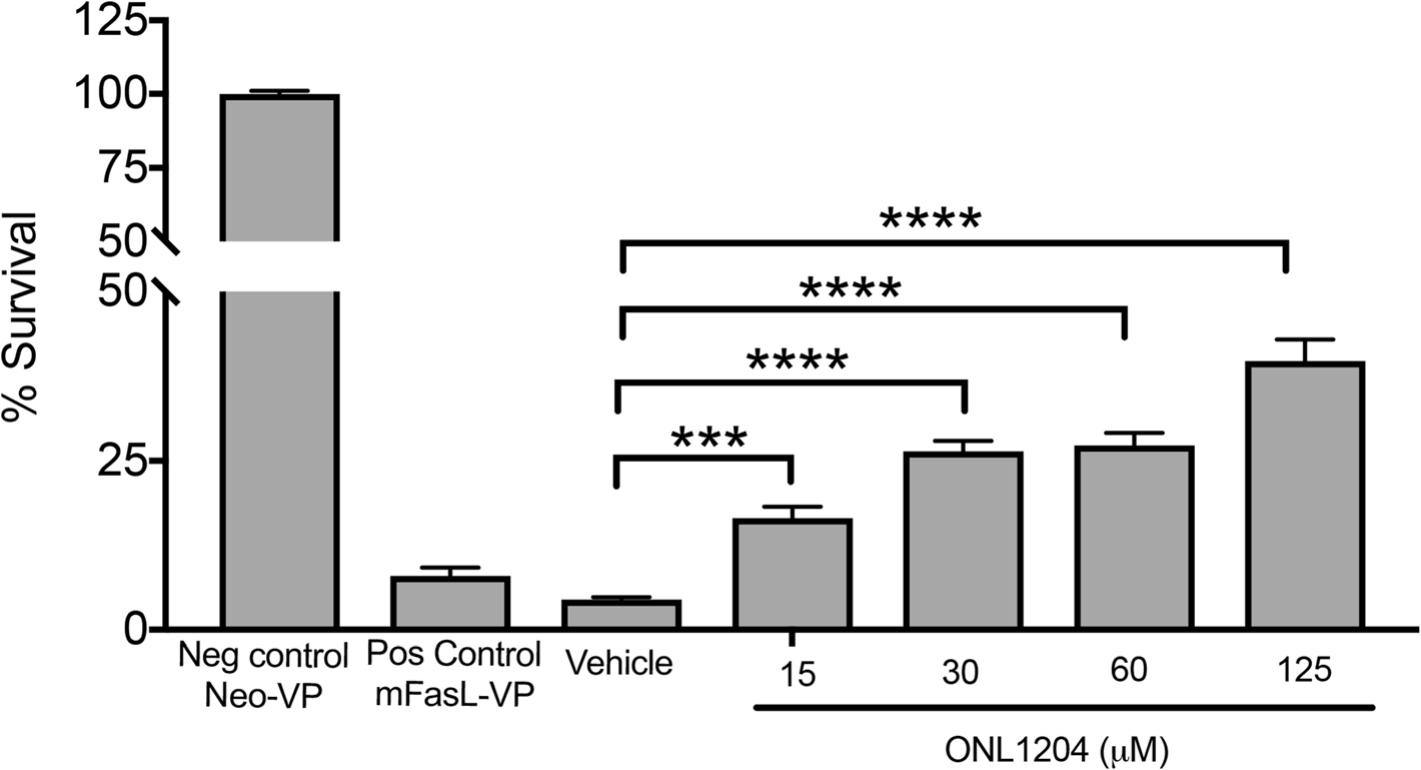
ONL1204 rescues A20 B lymphoma cells from FasL-mediated apoptosis. The ability of ONL1204 to inhibit FasL-mediated apoptosis of murine A20 B lymphoma cells was evaluated in vitro. Microvesicle preparations were isolated from transfected Neuro2a cells that expressed either murine mFasL (mFasL VP) or the vector control (Neo VP) as described previously [40]. A20 lymphoma cells were incubated for 4 h with increasing concentrations of ONL1204 or the vehicle control together with a 1:100 dilution of either mFasL VP or Neo VP and then cultured overnight in the presence of ^3^H-thymidine. Percent survival was assessed by ^3^H-thymidine incorporation using the formula (cpm of A20 cells cultured with mFasL VP + ONL1204 or vehicle)/(cpm of A20 cells incubated with media alone). A20 cells cultured with mFasL-VP alone served as the positive control (≈ 8% survival), while A20 cells cultured with neo-VP alone served as the negative control (≈ 100% survival). Data presented as % survival ± SEM. *N* = 6 per group, ***P < 0.001, ****P < 0.0001

We previously demonstrated that a small peptide antagonist of the Fas receptor (Met12) inhibits Fas-induced Caspase 8 activation and cell death of photoreceptors and retinal pigment epithelial cells in models of retinal detachment and NaIO_3_ model of oxidative stress respectively [34, 35]. In this study, we used a new derivative of Met12, ONL1204, with improved pharmaceutical properties. To confirm that ONL1204 blocks Fas death receptor signaling, we treated Fas+ murine A20 B lymphoma cells with membrane-FasL-expressing microvesicles in the presence of increasing concentrations of ONL1204 (Fig. 1). We previously demonstrated that microvesicles isolated from transfected Neuro2a cells expressing murine membrane-bound FasL (mFasL-VP) can serve as a cell-free source of mFasL that is highly efficient in killing Fas+ murine A20 B lymphoma cells [40]. Microvesicles isolated from Neuro2a cells transfected with the vector control (Neo-VP) do not express mFasL and serve as a negative control. Herein, A20 cells were treated with mFasL-VP at a 1:100 dilution for 4 h, and ^3^H-Thymidine incorporation indicated significant cell death with only 8.0% survival when compared to A20 cells incubated with media alone (Fig. 1). By contrast, no significant cell death was observed in A20 cells treated with Neo-VP, resulting in 100% survival when compared to A20 cells incubated with media alone. To determine whether ONL1204 could block apoptosis triggered by mFasL-VP, A20 cells were treated with mFasL-VP at a 1:100 dilution for 4 h in the presence of increasing concentrations of ONL1204 or vehicle control. Our results showed that FasL-induced apoptosis was inhibited by ONL1204 in a dose-dependent manner, while vehicle only had no effect (Fig. 1). These results demonstrate that ONL1204 can block activation of the Fas death receptor signaling pathway and prevent mFasL-induced apoptosis.

### Fas activation is required for death of RGCs and axon degeneration in an inducible mouse model of glaucoma

**Fig. 2:**
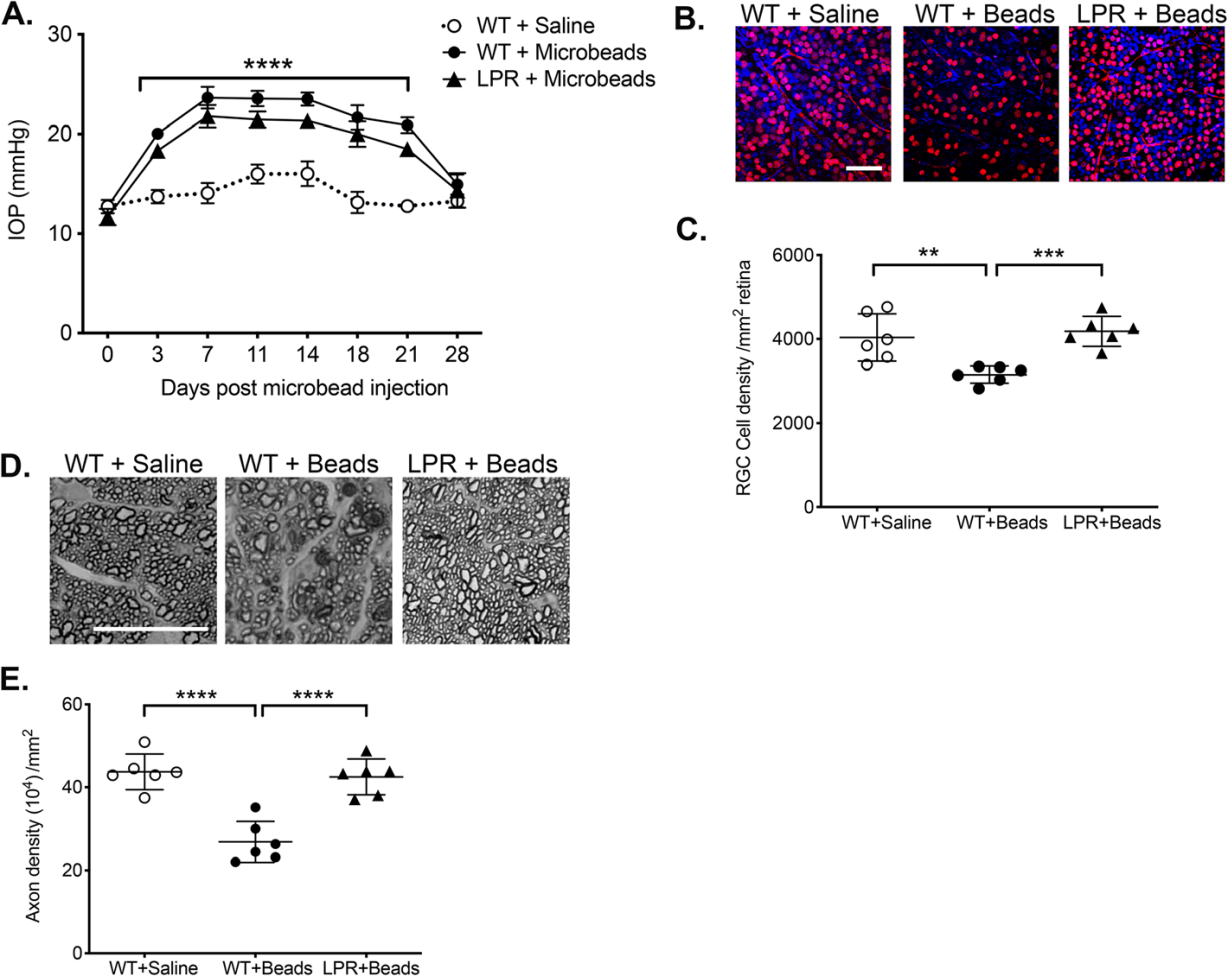
Fas signaling pathway is required for death of RGCs and loss of axons in microbead-induced model of glaucoma. **a** IOP measurements were taken by rebound tonometry in WT and Fas-deficient Fas^lpr^ mice injected with microbeads or saline. Data is presented as mean IOP ± SD, *N* = 6 mice per group. IOP was significantly elevated on days 3–21 in WT and LPR mice that received microbeads as compared with WT control mice receiving saline (****P < 0.0001). **b** Representative confocal images of retinal flatmounts isolated at 28 days post-microbead or saline injection and stained with an anti-Brn3a antibody (red, RGC-specific marker) and a DAPI nuclear stain (blue) (scale bar, 50 μm). **c** Quantification of Brn3a positive RGCs represented as the mean RGC density/mm^2^ retina ± SD. *N* = 6 per group, ***P<0.001, **P<0.01. **d** Representative photomicrographs of PPD-stained optic nerve cross-sections at 28 days post-microbead or saline injections (scale bar, 20 μm). **e** Quantification of healthy axons represented as the mean axon density (10^4^)/mm^2^ ON ± SD. *N* = 6 per group, ****P < 0.0001

Using genetically modified mice, we demonstrated previously that the membrane-bound form of FasL (mFasL) is neurotoxic and accelerates RGC death and axon degeneration in inducible and chronic mouse models of glaucoma [31, 36]. By contrast, overexpression of the soluble form of FasL (sFasL) via AAV-mediated gene delivery prevented RGC death and axon degeneration [31]. While these previous studies revealed opposing roles for mFasL and sFasL in the pathogenesis of glaucoma, the requirement of the Fas signaling pathway for the development of glaucoma was never demonstrated. Therefore, to determine whether Fas signaling was required for the development of glaucoma, we utilized a well-defined microbead-induced mouse model of elevated IOP to induce elevated IOP in C57BL/6J WT mice and Fas-deficient LPR mice (Fas^lpr)^) [41]. As previously described [31], a single anterior chamber injection of 15 μm polystyrene microbeads resulted in elevated IOP for up to 21 days in C57BL/6J WT as compared to saline controls (Fig. 2a). The IOP was monitored by rebound tonometry, and there was no significant difference in the time course or magnitude of the microbead-induced elevated IOP between Fas^lpr^ mice or C57BL/6J WT mice, indicating Fas signaling was not involved in the elevation of IOP. At 4 weeks post-microbead injection, RGC density was measured in retinal whole mounts stained with a RGC-specific anti-Brn3a antibody [37] (Fig. 2b, c) and axon density was measured in optic nerve sections stained with PPD [31] (Fig. 2d, e). Quantification of RGCs revealed a significant decrease in RGC density in microbead-injected WT mice as compared to saline-injected controls (Fig. 2c). However, in the absence of Fas signaling, the RGC density in microbead-injected Fas^lpr^ mice was equal to that of saline-injected controls (Fig. 2c). Similar results were observed in the optic nerve where Fas deficiency afforded complete protection of axons in microbead-injected Fas^lpr^ mice as compared to the microbead-injected C57BL/6 WT mice (Fig. 2d, e). Taken together, these results demonstrate that Fas signaling is *required* for the death of RGCs and loss of axons in the microbead-induced mouse model of glaucoma.

### Pre-elevated IOP treatment with ONL1204 protects RGCs and prevents axon degeneration in the microbead-induced model of glaucoma

**Fig. 3:**
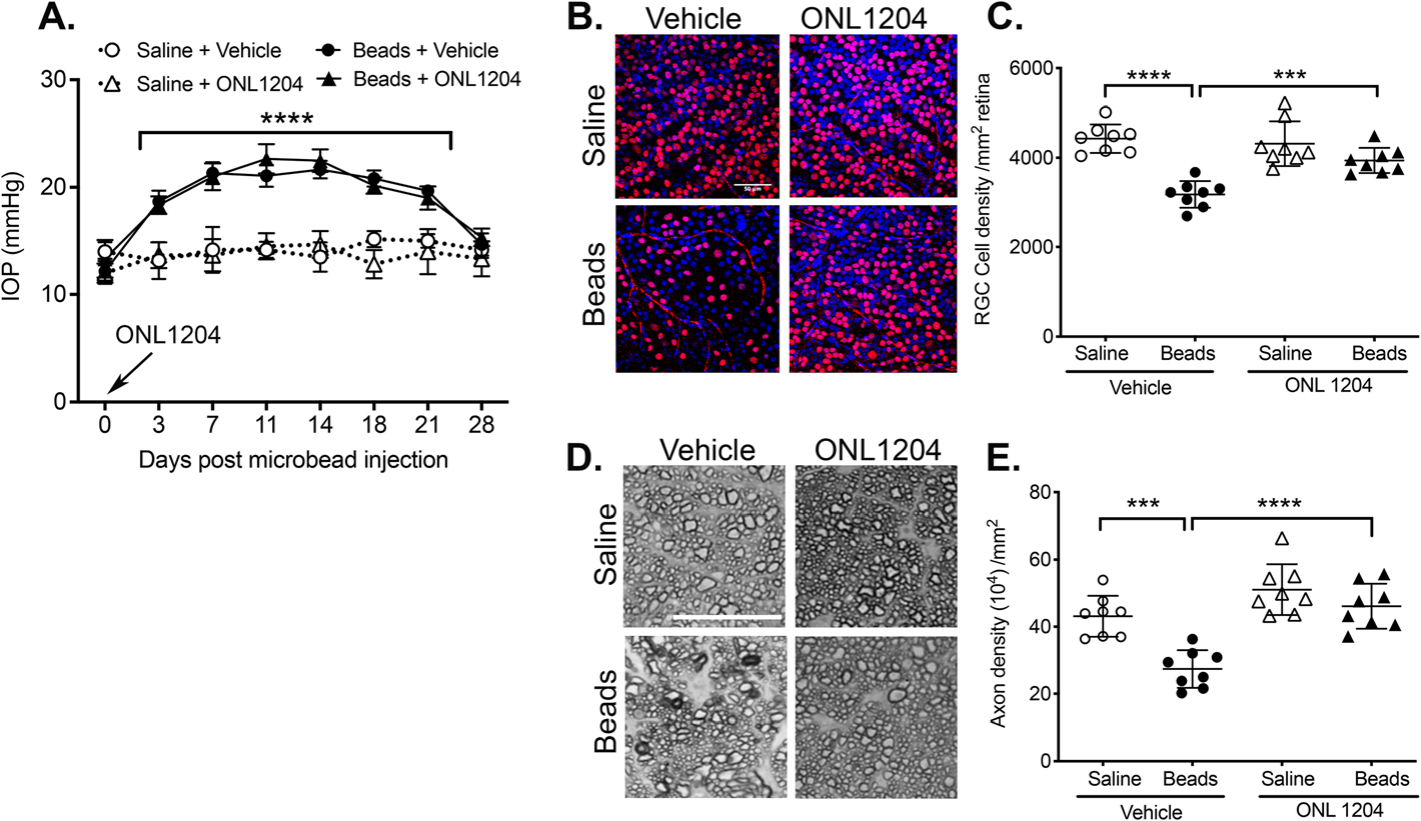
Pre-elevated IOP treatment with ONL1204 protects RGCs and prevents axon degeneration in microbead-induced model of glaucoma. WT C57BL/6J mice received an intravitreal injection of ONL1204 (2 μg/1 μl injection) or vehicle only, immediately followed by an anterior chamber injection of microbeads or saline (day 0). **a** IOP measurements were taken by rebound tonometry every 3–4 days. Data is presented as mean IOP ± SD, *N* = 8 mice per group. IOP was significantly elevated on days 3–21 in microbead-injected WT mice treated with ONL1204 or vehicle when compared to saline-injected WT controls treated with ONL1204 or vehicle (****P < 0.0001). **b** Representative confocal images of retinal flatmounts isolated at 28 days post-microbead or saline injection and stained with an anti-Brn3a antibody (red, an RGC-specific marker) and a DAPI nuclear stain (blue) (scale bar, 50 μm). **c** Quantification of Brn3a-positive RGCs represented as the mean RGC density/mm^2^ retina ± SD. *N* = 8 per group, ***P < 0.001, ****P < 0.0001. **d** Representative photomicrographs of PPD-stained optic nerve cross-sections at 28 days post-microbead or saline injections (scale bar, 20 μm). **e** Quantification of healthy axons represented as the mean axon density (10^4^)/mm^2^ ON ± SD. *N* = 8 per group, ***P < 0.001, ****P < 0.0001

To determine the neuroprotective potential of Fas-receptor inhibition in glaucoma, we pretreated C57BL/6J WT mice with ONL1204, prior to induction of elevated IOP. In this study, C57BL/6J WT mice received an intravitreal injection of ONL1204 (2 μg/μl) or vehicle only, immediately preceding injection of microbeads or saline. IOPs were monitored every 3–4 days by rebound tonometry and revealed no significant difference in the time course or magnitude of the microbead-induced elevated IOP between mice treated with ONL1204 or vehicle only, indicating ONL1204 did not affect IOP (Fig. 3a). Quantification of RGCs at 4 weeks post-microbead injection revealed a significant decrease in RGC density in microbead-injected vehicle-treated mice as compared to the saline-injected controls (Fig. 3b, c). However, pretreatment with ONL1204 was neuroprotective, and the RGC density in microbead-injected ONL1204-treated mice was equal to the RGC density in the saline-injected controls (Fig. 3b, c). Similar results were observed in the optic nerve with a significant decrease in axon density detected in microbead-injected, vehicle-treated mice when compared to saline-injected controls, while pretreatment with ONL1204 afforded complete protection of axons with axon density in microbead-injected ONL1204-treated mice equal to that of the saline-injected controls (Fig. 3d, e). Taken together, these results demonstrate that pretreatment with the Fas inhibitor, ONL1204, prior to elevated IOP provides significant neuroprotection to both RGCs and their axons in the microbead-induced mouse model of glaucoma.

### Post-elevated IOP treatment with ONL1204 protects RGCs and prevents axon degeneration in the microbead-induced model of glaucoma

**Fig. 4:**
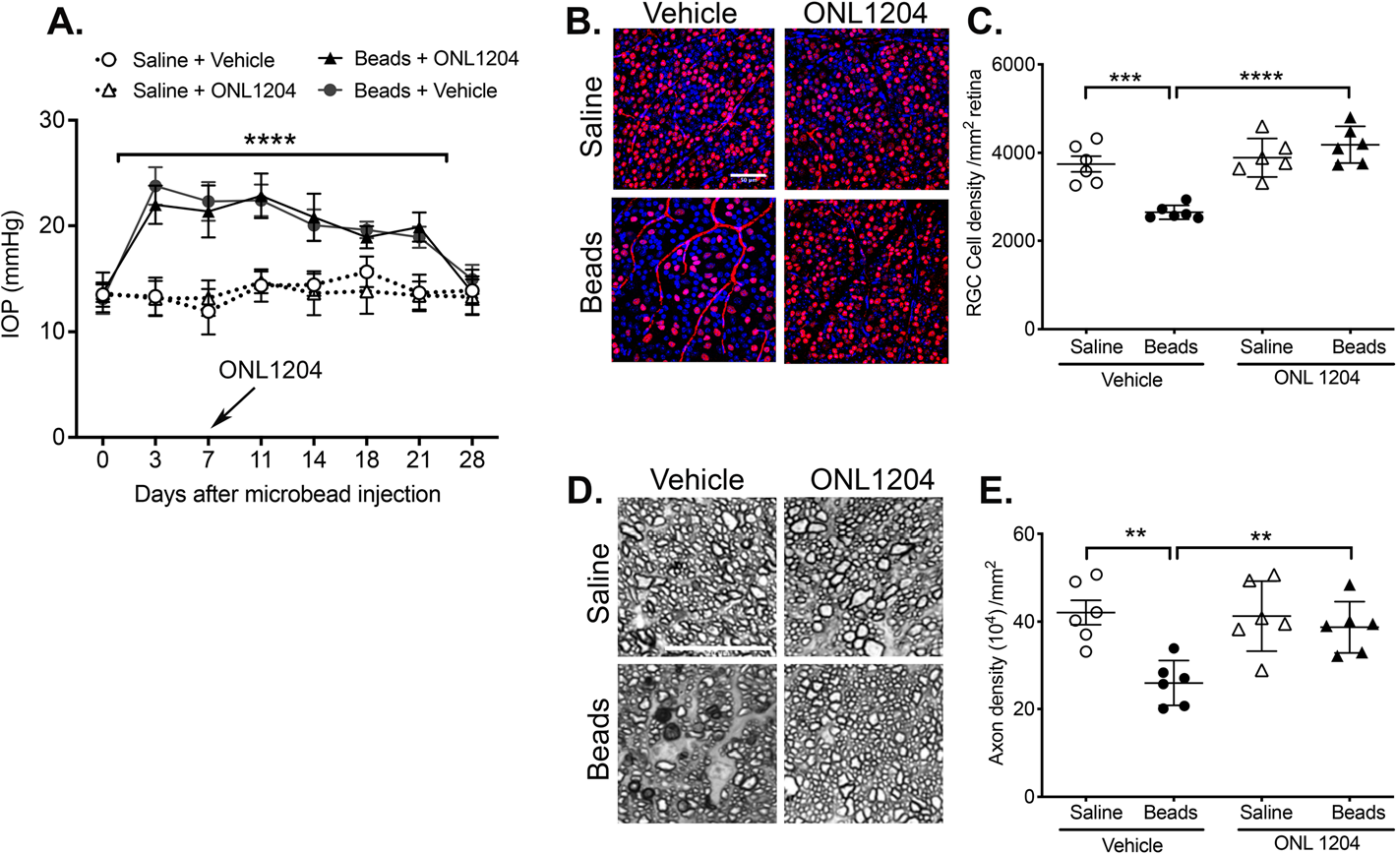
Post-elevated IOP treatment with ONL1204 protects RGCs and prevents axon degeneration in microbead-induced model of glaucoma. WT C57BL/6J mice received an intravitreal injection of ONL1204 (2 μg/1 μl injection) or vehicle at 7 days post-anterior chamber injection of microbeads or saline. **a** IOP measurements were taken by rebound tonometry every 3–4 days. Data is presented as mean IOP ± SD, *N* = 6 mice per group. IOP was significantly elevated on days 3–21 in microbead-injected WT mice treated with ONL1204 or vehicle when compared to saline-injected WT controls treated with ONL1204 or vehicle (****P < 0.0001). **b** Representative confocal images of retinal flatmounts isolated at 28 days post-microbead or saline injection and stained with an anti-Brn3a antibody (red, an RGC-specific marker) and a DAPI nuclear stain (blue) (scale bar, 50 μm). **c** Quantification of Brn3a-positive RGCs represented as the mean RGC density/mm^2^ retina ± SD. *N* = 6 per group (***P < 0.001, ****P < 0.0001). **d** Representative photomicrographs of PPD-stained optic nerve cross-sections at 28 days post-microbead or saline injections (scale bar, 20 μm). **e** Quantification of healthy axons represented as the mean axon density (10^4^)/mm^2^ ON ± SD. *N* = 6 per group, **P < 0.01

While pretreatment with ONL1204 provided significant neuroprotection in the microbead-induced mouse model of glaucoma, the more clinically relevant question is whether treatment with ON1204 can provide neuroprotection even when administered after detection of elevated IOP, as this would be the time at which glaucoma patients would most likely receive treatment. To answer this question, C57BL/6J WT mice received an anterior chamber injection of microbeads or saline, and at 7 days post-microbead injection, all mice receive an intravitreal injection of ONL1204 or vehicle alone. IOPs were monitored every 3–4 days by rebound tonometry, verifying that IOP was elevated prior to intravitreal injection of the drug or vehicle. The IOP data revealed no significant difference in the time course or magnitude of the IOP between mice treated with ONL1204 or vehicle at 7 days post-microbead injection (Fig. 4a). At 4 weeks post-microbead injection, quantification of RGCs revealed significant preservation of RGCs in the ONL1204-treated mice when compared to mice treated with vehicle alone (Fig. 4b, c). Significant protection of axons was also observed with axon density in ONL1204-treated mice equivalent to the axon density in saline-treated controls (Fig. 4d, e). In conclusion, these data demonstrate that inhibition of Fas activation provides significant protection to both the RGCs and axons, even when administered after elevated IOP.

### ONL1204-mediated neuroprotection correlates with reduced activation of Iba1+ microglia and/or infiltrating macrophages

**Fig. 5:**
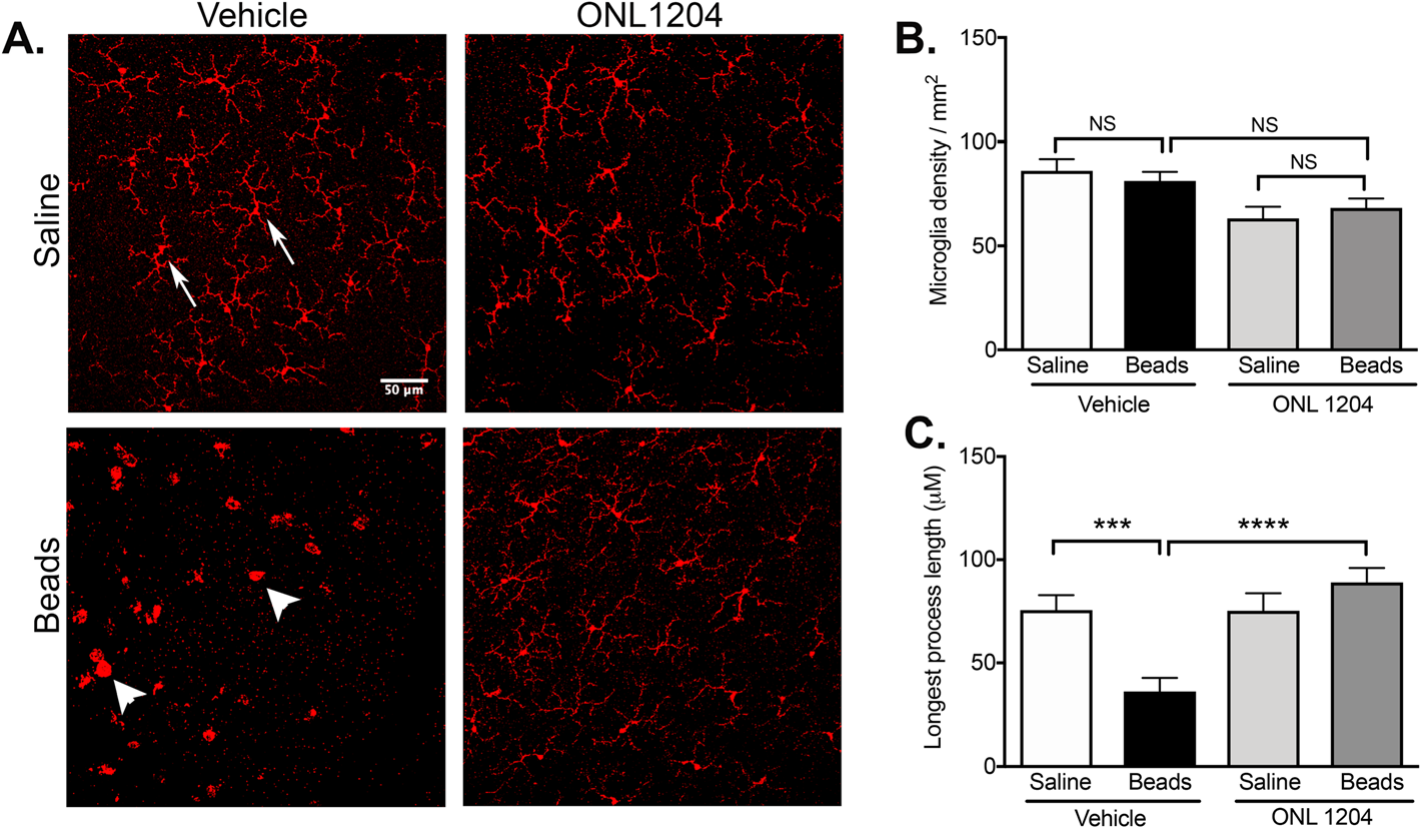
Inhibition of Fas signaling prevents activation of retinal microglia following elevated IOP. WT C57BL/6J mice received an intravitreal injection of ONL1204 (2 μg/1 μl injection) or vehicle just prior to an anterior chamber injection of microbeads or saline (day 0). **a** Representative confocal images of retinal flatmounts isolated at 28 days post-microbead or saline injection and stained with an anti-Iba1 antibody (red, microglia/macrophage marker). Iba1+ cells in the saline-injected WT mice treated with vehicle or ONL1204 displayed a quiescent phenotype with dendritic morphology (white arrow), while Iba1+ cells in microbead-injected WT mice treated with vehicle display a more activated phenotype, with amoeboid morphology (white arrowhead) that is inhibited in microbead-injected mice treated with ONL1204 (scale bar, 50 μm). **b** Quantification of Iba1+ cells in the GCL/IPL revealed no significant difference between any groups at 28 days post-microbead injection. **c** Morphometric analysis was performed on Iba1+ cells in the GCL/IPL (60 cells per retina), and the longest process length measured from the edge of the cell body (in micrometer) was used to quantitate microglia activation as previously described. Data presented as the mean microglia density/mm^2^ ± SD and the mean longest process length in μM ± SD. *N* = 3–4 per group, ***P < 0.001, ****P < 0.0001

In human and experimental models of glaucoma, activated microglia are detected in the optic nerve head and retina [14–19], and blocking microglia activation with minocycline [14, 20], anti-TNF [22, 23], or irradiation [42] prevents death of RGCs and axon degeneration. While triggering the Fas receptor is best known for inducing apoptosis, we previously demonstrated that accelerated death of RGCs in mice that only express the membrane form of FasL (mFasL) correlated with increased activation of retinal microglia, suggesting that FasL mediates both RGC apoptosis and glial activation [36]. To determine whether ONL1204-mediated neuroprotection correlates with inhibition of microglia activation in the neural retina, C57BL/6J WT mice were pretreated with ONL1204 just prior to the anterior chamber injection of microbeads. At 28 days post-microbead injection, retinal whole mounts were stained with Iba1 (microglia/macrophage marker). Retinal microglia are located in the ganglion cell layer (GCL), inner plexiform layer (IPL), and outer plexiform layer (OPL). However, we did not detect any changes in the microglia in the OPL during the development of glaucoma in our model system, and since glaucoma specifically targets the RGCs and their axons, we focused our analysis on microglia in the GCL and IPL of the retina. Representative confocal images from saline control groups treated with vehicle or ONL1204 reveal Iba1+ cells with a quiescent phenotype and dendritic morphology (Fig. 5a), while confocal images from microbead-injected mice treated with vehicle only reveal Iba1+ cells with an activated phenotype and amoeboid morphology (Fig. 5a). By contrast, the Iba1+ cells in retinal whole mounts prepared from microbead-injected mice pretreated with ONL1204 maintained a quiescent phenotype with dendritic morphology, similar to that observed in the saline-injected control groups (Fig. 5a). Quantification of microglia density in the GCL/IPL revealed no significant difference in absolute number of Iba1+ cells at 28 days post-microbead injection between all groups (Fig. 5b). However, quantification of microglia activation, using the measurement of the longest process length as previously described [39], revealed a significant shortening of the cell process length in Iba1+ cells from microbead-injected mice treated with vehicle as compared to Iba1+ cells from microbead-injected mice treated with ONL1204 (Fig. 5c). These data indicate that Fas activation mediates both RGC apoptosis and microglia activation.

### ONL1204-mediated neuroprotection correlates with a significant reduction in inflammatory cytokines and chemokines

**Fig. 6:**
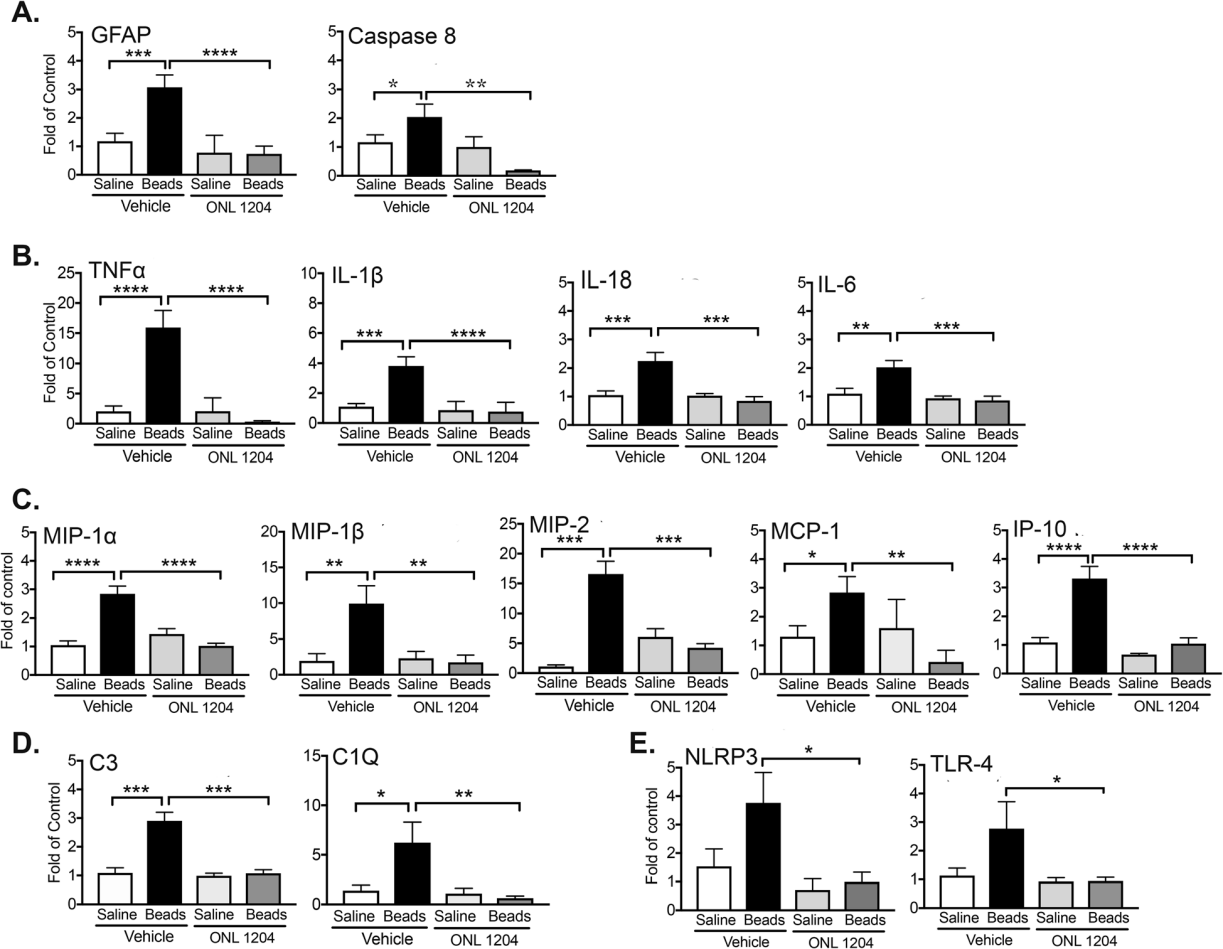
Inhibition of Fas signaling prevents the induction of multiple inflammatory pathways implicated in the pathogenesis of glaucoma. At 28 days post-microbead or saline injection, quantitative PCR was performed on neural retina isolated from saline- and microbead-injected WT mice treated (on day 0) with ONL1204 or vehicle to assess the expression of several proinflammatory genes associated with human and/or experimental models of glaucoma: **a** Caspase 8 and GFAP, **b** proinflammatory cytokines (TNFα, IL-1β, IL-18, and IL-6), **c** proinflammatory chemokines (MIP-1α, MIP-1β, MIP-2, MCPI, and IP10), **d** complement components C3 and C1Q, and **e** NLRP3 and TLR4. The threshold cycle values of each gene of interest were normalized to the geometric mean of two housekeeping genes (B2-microglobulin and peptidylpropyl isomerase A) and compared with the saline + vehicle control group using the comparative C method (ΔΔC). Data are presented as fold change of control ± SEM. *N* = 6 per group, *P < 0.05, **P < 0.01, ***P < 0,001, ****P < 0.0001

In human and experimental glaucoma, multiple inflammatory pathways have been implicated in the pathogenesis of disease, including the Toll-like receptor signaling pathway [43], the inflammasome pathway [44–47], the TNFα pathway [22, 23, 48–50], and the complement cascade [51–53]. While triggering of the Fas receptor is best known for inducing apoptosis through the activation of caspase-8, activated caspase-8 can also induce the production of pro-inflammatory mediators [54–57]. Moreover, caspase-8 activation has also been linked to microglia activation [58] and inflammation in experimental models of glaucoma and inhibiting caspase-8 blocked inflammation and prevented death of RGCs [45]. To explore the role of Fas activation in triggering inflammation in glaucoma, we pretreated C57BL/6J WT mice with ONL1204 just prior to injection of microbeads, and at 28 days post-microbead injection, the neural retina was isolated, and qPCR was performed to assess the expression of several proinflammatory genes associated with human and/or experimental models of glaucoma. We first examined the gene expression of caspase-8, which plays an essential role in Fas receptor signaling cascades that induces both apoptosis and cytokine production [55], as well as GFAP as a measure of glial activation. The qPCR analysis revealed a significant induction in both GFAP and Caspase-8 in microbead-injected mice treated with vehicle only, as compared to saline-treated controls (Fig. 6a). We then examined the gene expression of several proinflammatory cytokines (TNFα, IL-1β, IL-6, and IL-18) (Fig. 6b) and chemokines (MIP-1α, MIP-1β, MIP-2, MCPI, and IP10) (Fig. 6c) that have been implicated in human and experimental models of glaucoma [43, 48, 59–61]. The qPCR analysis revealed significant induction of each of these genes in the retina of microbead-injected mice treated with vehicle only as compared to the saline-treated controls (Fig. 6b, c). By contrast, pretreatment with ONL1204 prevented the induction of each of these genes and gene expression was equivalent to the saline-treated control (Fig. 6b, c). Additionally, key mediators of the Toll-like receptor pathway, inflammasome pathway, and complement cascade that have been identified in human and experimental glaucoma were also examined, specifically TLR4 [43, 44], NLRP3 [44, 45, 48], and complement components C3 and C1Q [53, 62, 63]. Similar to the proinflammatory cytokines and chemokines, gene expressions of C3, C1Q, TLR4, and NLRP3 were all significantly induced at 28 days post-microbead injection in the retina of microbead-injected mice treated with vehicle only as compared to saline-treated controls (Fig. 6d, e). However, the induction of each of these genes was inhibited in microbead-injected mice that were pretreated with ONL1204 (Fig. 6d, e). Together, these results suggest that Fas activation is upstream to multiple inflammatory pathways that have been implicated in glaucoma and blocking Fas activation with ONL1204 prevents RGC apoptosis, as well as microglia activation and the induction of neurodestructive inflammation.

## Discussion

In this study, we evaluated the neuroprotective effect of ONL1204, a novel small peptide inhibitor of the Fas receptor, in a microbead-induced mouse model of elevated IOP. We previously demonstrated that apoptosis of RGCs in both inducible and chronic mouse models of glaucoma was dependent upon the FasL-Fas signaling pathway [31, 36]. While several studies have implicated the proinflammatory cytokine TNFα as the critical link between elevated IOP and death of RGCs in glaucoma [22, 23, 48, 49], Nakazawa et al. demonstrated in a laser-induced mouse model of ocular hypertension that TNFα does not directly kill RGCs, but rather RGC death is dependent upon TNFR2-mediated activation of microglia [23]. Our laboratory went on to demonstrate that TNFα increased expression of FasL on microglia in the glaucomatous retina and that the membrane-bound form of FasL was the key effector of RGC apoptosis in an inducible mouse model of glaucoma [36]. However, it has become increasingly apparent that in addition to apoptosis, Fas-mediated signaling can also induce the release of proinflammatory cytokines and promote inflammation [40, 64–66]. Using our novel Fas inhibitor, ONL1204, in an inducible mouse model of glaucoma, we show that blocking Fas activation prevents axon degeneration and the death of RGCs, as well as the activation of microglia and the induction of multiple inflammatory genes previously implicated in both experimental and human glaucoma. Importantly, many of the cytokines and chemokines we have now quantified in the glaucomatous eyes are the same proinflammatory molecules that we previously found to be produced by FasL-treated macrophages [40]. Moreover, the data presented herein provide proof of principle that treatment with ONL1204 effectively blocks Fas activation and affords significant neuroprotection to RGCs and their axons in an experimental model of glaucoma. Having identified the FasL-Fas signaling pathway as an essential pathway in the pathogenesis of glaucoma, the first aim of the present study was to evaluate the neuroprotective effect of our novel Fas inhibitor, ONL1204, in the microbead-induced mouse model of glaucoma. The ONL1204 inhibitor is a new derivative of Met12, a small peptide that we showed could inhibit Fas activation and subsequent apoptosis of photoreceptors and retinal pigment epithelial cells in models of retinal detachment and retinal degeneration [34, 35]. Through assessment of axon degeneration and RGC survival, our results provide proof-of-principal that ONL1204 can provide robust neuroprotection in an inducible mouse model of glaucoma, even when administered after the detection of elevated IOP. In addition, we found that ONL1204-mediated neuroprotection correlates with significantly reduced activation of retinal microglia and no significant induction of proinflammatory genes implicated in both human and experimental glaucoma. These data support our hypothesis that in glaucoma, Fas activation is a critical mediator of RGC apoptosis, as well as microglial activation and neuroinflammation.

The results of this present study are in agreement with our previous work using an AAV2-mediated gene therapy approach to deliver soluble FasL, considered an antagonist of the proapoptotic and proinflammatory membrane form of FasL [31]. Overexpression of sFasL using the AAV2-mediated gene therapy approach prevented axon degeneration and death of RGCs in both inducible and chronic mouse models of glaucoma, and this neuroprotection correlated with an inhibition of Müller glia activation and induction of inflammatory mediators [31]. Taken together, the previous results of our sFasL-AAV2 study combined with results of the present study with ONL1204 strongly support the value of Fas inhibition as an approach to neuroprotection in glaucoma, both in preserving RGC viability and in preventing neuroinflammation.

Neuroinflammation has long been associated with chronic neurodegenerative diseases such as Alzheimer’s and Parkinson’s [67–69]. However, while glial activation and inflammatory cytokines have been detected in the optic nerve head and retina of human [17, 19, 48] and experimental models of glaucoma [20, 21, 60, 70], the specific impact of glial activation and neuroinflammation on the development and/or progression of glaucoma is not well understood. In human and experimental models of glaucoma, activated microglia are detected in the ONH and retina [14, 16, 17, 19, 20]. Microglia are the resident innate immune cells of the retina and optic nerve and are responsible for normal maintenance of neuronal tissue, as well as a local response to injury. However, in retinal degenerative diseases, chronic microglia activation has been linked to retinal damage and neuronal apoptosis [71] and the extent of microglia activation in the ONH coincides with the severity of axon degeneration [14, 20, 21]. Moreover, Barres and colleagues demonstrate in the CNS that neurotoxic astrocytes are induced by activated microglia [72] and blocking microglial activation with minocycline [14, 20] or anti-TNF [22, 23] prevents axon degeneration and death of RGCs suggesting that activated microglia are the driving force behind the axon degeneration and death of RGCs in glaucoma. However, the molecular mechanism(s) that mediate microglia reactivity in glaucoma have not yet been defined.

While the specific trigger(s) of neuroinflammation in glaucoma remains poorly defined, a number of key inflammatory pathways have been implicated in the pathogenesis of glaucoma and are common to both human and animal models of glaucoma. These pathways include the complement cascade [53, 62, 63], Toll-like receptor pathway [43, 44], TNFα pathway [22, 23, 48–50], and inflammasome pathway [44–47]. Using the microbead-induced mouse model of glaucoma, we also show an induction of genes associated with each of these pathways at 4 weeks post-microbead injection, specifically C3 and C1Q (complement cascade), TLR4 (Toll-like receptor pathway), TNFα (TNFα pathway), and NLRP3 (inflammasome pathway). However, treatment with ONL1204 completely abrogated the induction of each of these genes, indicating that Fas activation is upstream to these pathways and plays a central role in mediating neuroinflammation in glaucoma. In addition, the induction of these inflammatory genes correlated with a significant increase in the number of activated, amoeboid-shaped Iba1+ cells in the retina and treatment with ONL1204 completely abrogated the activation of Iba1+ cells in the retina with the Iba1+ cells displaying a homeostatic, dendritic phenotype indistinguishable from the non-glaucoma controls. Together, these data indicate that blocking Fas signaling prevents microglial activation and the development of neuroinflammation.

However, the Fas receptor is expressed on multiple retinal cell types, including astrocytes, RGCs, Mueller cells, microglia, and retinal pigment epithelial cells [7, 31, 36, 73]. Therefore, additional studies in which the Fas receptor is deleted from specific cell types will be necessary to determine which Fas receptor-positive cell(s) actually drive(s) the development of neuroinflammation in glaucoma. Moreover, Fas mediates both apoptotic and inflammatory pathways and it is not possible from the current studies to determine the extent to which Fas-mediated apoptosis and/or Fas-mediated inflammation contributes to axon degeneration and death of RGCs in glaucoma. Yet, previous therapeutic approaches that specifically targeted the apoptotic pathway alone resulted in neuroprotection of the RGC soma but failed to prevent axon degeneration [11, 12], suggesting the robust neuroprotective effect afforded by ONL1204 is dependent upon the ability of ONL1204 to antagonize both Fas-mediated apoptosis of RGCs and Fas-mediated activation of retinal microglia and induction of neuroinflammation. Additional studies in which the Fas receptor is specifically knocked out in RGCs or glial cells (microglia, astrocytes, and Mueller cells) are necessary to determine if the neuroprotective effects of ONL1204 are mainly driven by modulating the inflammatory response of retinal glial cells or preventing FasL-induced apoptosis of RGCs.

While triggering of the Fas receptor is known for inducing apoptosis through the activation of caspase-8, activated caspase-8 can also induce the production of pro-inflammatory mediators [44, 55, 57, 74]. Although activation of IL-1β and IL-18 is most often thought to be inflammasome-dependent, we recently demonstrated that Fas can mediate IL-1β and IL-18 maturation via a caspase-8-dependent inflammasome-independent mechanism [55]. In addition, caspase-8 activation has been linked to inflammation in experimental models of glaucoma and inhibition of caspase-8 blocks inflammation and prevents death of RGCs [44, 45]. Yet, similar to our findings presented herein, the previous caspase-8 studies were unable to determine the extent to which caspase-8-mediated inflammation and/or caspase-8-mediated apoptosis contributed to axon degeneration and RGC apoptosis. However, while caspase-8-mediated inflammation can be triggered by the Fas-receptor [55, 74], TRAIL receptor [75], and Toll-like receptors (TLRs) [76, 77], we demonstrate herein that specifically blocking Fas activation in the microbead-induced mouse model of glaucoma inhibits the induction of caspase-8, retinal microglia activation, and the induction of proinflammatory genes, indicating the TRAIL- and TLR-mediated pathways are downstream of the FasL-Fas pathway. Moreover, determining the extent to which Fas-mediated inflammation and/or apoptosis contributes to axon degeneration and death of RGCs in glaucoma will require the uncoupling the Fas-mediated apoptosis and Fas-mediated inflammatory pathways and this will be the focus of our future studies.

As a complex multifactorial disease, we predict the most successful neuroprotective therapy for glaucoma will have to impact multiple pathways and the data presented herein strongly support pursuing the FasL-Fas signaling pathway as an optimal target for successful neuroprotection in glaucoma. Specifically blocking Fas activation in this present study resulted in significant inhibition of glial activation, neuroinflammation, and RGC death. In the normal eye, the FasL-Fas signaling pathway plays an essential role in the maintenance of ocular immune privilege where inflammation is tightly regulated [28, 78, 79]. However, it has become increasingly clear that immune privilege is not simply established through suppression of all immune responses, but rather through modulation of the immune responses in a way that provides immune protection to the delicate tissues of the eye while limiting the development of destructive inflammation. FasL is constitutively expressed in the immune-privileged eye where the membrane form of FasL is the active form, inducing apoptosis of infiltrating Fas+ immune cells [28, 78, 79]. However, because the Fas receptor is ubiquitously expressed on multiple cell types throughout the eye, the cleavage or shedding of mFasL acts to limit the expression of mFasL and prevent the killing of healthy Fas+ bystander cells [80, 81]. However, in glaucoma, the cleavage or shedding of mFasL is significantly reduced resulting in a significant decrease in release of sFasL and concomitant increase in the expression of mFasL that correlates with the apoptosis of Fas+ RGCs [31]. Therefore, we propose that either treatment with sFasL, as we previously demonstrated [31], or treatment with a Fas inhibitor as shown in this present study will work to (i) block the proapoptotic and proinflammatory activity of mFasL, (ii) promote restoration of the ocular immune-privileged environment, and (iii) support the return of the activated retinal microglia to their original homeostatic phenotype.

## Conclusions

In summary, our data provide proof-of-principal that treatment with the small peptide inhibitor of the Fas receptor, ONL1204, provides significant protection of the RGC soma and their axons in an inducible mouse model of glaucoma. In addition, the studies presented herein demonstrate the requirement of Fas activation in both the death of RGCs and axon degeneration, as well as the activation of retinal microglial and induction of neuroinflammation in the development of glaucoma. Future studies will be aimed at identifying the critical source of FasL and uncoupling the Fas-mediated apoptosis and Fas-mediated inflammatory pathways in order to determine the extent to which inflammation versus apoptosis contributes to the development and progression of glaucoma.

## Data Availability

The datasets generated during and/or analyzed during the current study are available from the corresponding author upon reasonable request.

## Abbreviations

FasL: Fas ligand
GCL: Ganglion cell layer
IOP: Intraocular pressure
mFasL: Membrane-bound Fas ligand
ON: Optic nerve
ONH: Optic nerve head
PPD: Paraphenylenediamine
RGCs: Retinal ganglion cells
sFasL: Soluble Fas ligand
